# Distortion of Population Statistics due to the Use of Different Methodological Approaches to the Construction of Genomic DNA Libraries

**DOI:** 10.32607/actanaturae.11898

**Published:** 2023

**Authors:** F. S. Sharko, K. V. Zhur, V. A. Trifonov, E. B. Prokhortchouk

**Affiliations:** Laboratory of vertebrate genomics and epigenomics, Federal Research Centre “Fundamentals of Biotechnology” of the Russian Academy of Sciences, Moscow, 119071 Russian Federation; Institute for the History of Material Culture of the Russian Academy of Sciences, Saint Petersburg, 191186 Russian Federation

**Keywords:** ancient DNA, ADMIXTURE, UDG

## Abstract

Several different methods of DNA library preparation for paleogenetic studies are now available. However, the chemical reactions underlying each of them can affect the primary sequence of ancient DNA (aDNA) in the libraries and taint the results of a statistical analysis. In this paper, we compare the results of a sequencing of the aDNA libraries of a Bronze Age sample from burials of the Caucasian burial ground Klady, prepared using three different approaches: (1) shotgun sequencing, (2) strategies for selecting target genomic regions, and (3) strategies for selecting target genomic regions, including DNA pre-treatment with a mixture of uracil-DNA glycosylase (UDG) and endonuclease VIII. The impact of the studied approaches to genomic library preparation on the results of a secondary analysis of the statistical data, namely F4 statistics, ADMIXTURE, and principal component analysis (PCA), was analyzed. It was shown that preparation of genomic libraries without the use of UDG can result in distorted statistical data due to postmortem chemical modifications of the aDNA. This distortion can be alleviated by analyzing only the single nucleotide polymorphisms caused by transversions in the genome.

## INTRODUCTION

The Russian Federation is a rich source of archaeological material for paleogenetic studies. Materials from Russia have been used in one way or another in almost all discoveries that have involved ancient DNA (aDNA): the discovery of the Denisovan man [1], the Ancient North Eurasians (ANE) population [2], Eastern hunter-gatherers (EHG) [3], and the population of Yamnik [4]. Yet, paleogenetic studies sited in Russia have had limited success, which has mostly involved the donation of bone material [5]. However, a number of interdisciplinary teams have recently been established. These teams are focused on comprehensive studies: expeditionary finds, synthesis of archaeological and paleogenetic data, and the generation of new historical hypotheses. This work summarizes the methodological approaches tested at the Federal Research Center "Fundamentals of Biotechnology" of the Russian Academy of Sciences and provides the most effective algorithms for the construction of genomic libraries that can be used by other laboratories in the field.

Ancient DNA sequence analysis has become a powerful tool to study ancient human populations [6, 7, 8]. However, there is a number of difficulties associated with postmortem degradation of genetic material due to the action of endogenous nucleases, random hydrolysis, and oxidation. The most common aDNA damage is deamination of cytosine residues, i.e. cleavage of the amino group from the nucleobase, followed by the formation of uracil residues, which, in turn, are converted to thymine during the polymerase chain reaction at the stage of preparation of DNA fragment libraries [9]. As a result, when sequencing libraries of aDNA fragments, researchers observe substitutions in either the C→T direction at the 5’ end of the DNA molecule or G→A direction at the 3’ end, depending on the sample preparation protocol used. The presence of these substitutions (false nucleotides) reduces the accuracy of mapping reads to the reference sequence, meaning that the reads containing non-reference alleles are less likely to be mapped than those containing reference alleles [6].

The total number of false nucleotides present in a reconstructed genome depends on the amount of sequencing data obtained and postmortem DNA damage and whether or not DNA was pre-treated with a mixture of uracil-DNA glycosylase (UDG) and endonuclease VIII (the mixture makes it possible to remove uracil with the formation of a single-nucleotide gap) during the preparation of genomic libraries [10]. Reconstructed ancient genomes usually contain sequences with both genuine and artefactual variants, which can affect the allele frequency analysis and determination of the population structure [6].

Analysis of ancient genome databases (Allen Ancient DNA Resource, https://reich.hms.harvard.

Population structure analysis plays an important role in human evolutionary genetics, which makes it possible to characterize genetic variability [12]; i.e. the presence of different levels of genetic relationship between subgroups in the population. For instance, this is important for establishing the time of divergence between populations originating from different geographic locations [13, 14]. In order to solve this task mathematically, a formal assumption of the existence of so-called ancestral populations that gave rise to the analyzed groups is made. These ancestral populations are a mathematical abstraction; they are characterized by specific allele frequencies, and the contribution of these frequencies to real samples allows one to make brief visual summaries illustrating the presence of a population structure in the sample.

Genetic clustering algorithms implemented in such tools as STRUCTURE [15] and ADMIXTURE [16] are widely used for the characterization of individual samples and populations based on genetic data. ADMIXTURE effectively estimates individual ancestries by computing maximum likelihood estimates in a parametric model. This model states that the genotype n_ij_ for individual i at single nucleotide polymorphism (SNP) j is a number of type "1" alleles observed. Given K ancestral populations, the success likelihood in binomial distribution n_ij_ ~ Bin(2, p_ij_) is a function of the fraction q_ik_ of ancestry i for population k and the frequency fkj for allele 1 in population k. ADMIXTURE maximizes the log-likelihood of a model using a block relaxation algorithm:

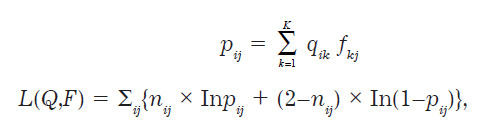



where q_ik_ and f_kj_ comprise the matrices Q and F, respectively [17].

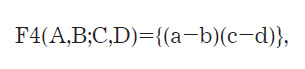



where A, B, C, and D are populations, while a, b, c, and d are the corresponding frequencies.

The result is considered statistically significant if the absolute value of the z score for the distribution of F4-statistics elements is > 3. In paleogenetics, it is often impossible to obtain populations frequencies due to the lack of a sufficient number of samples. In this case, the ABBA-BABA test is used as an F4-statistics analogue to genotype individual genomes. To be able to establish the likelihood that ABBA-BABA test values differ from zero, the absolute value of the z score should be > 3, while ABBA-BABA test values for uniform genomic windows are used as sample elements [18]. If one of the populations tested using F4 (or the ABBA-BABA test in the case of genotypes) is historically, morphologically, or genetically very distant from the studied groups (outgroup), then the intuitive explanation of the non-zero F4-statistics comes down to evaluating the contribution of the population located in the same branch with the outgroup to one of the two populations in the other graph branch.

If we present the genome as a vector with genotype values as coordinates that can be either 1, 0, or -1 (which stand for homozygotes AA and BB and heterozygote AB, respectively), then genotyped samples can be presented as a set of multidimensional (based on the total number of SNPs identified) vectors that can be projected onto lower dimensional spaces. PCA is one of the projection methods; it makes it possible to visualize relative to the position of objects. In particular, in ethnogeographic studies, pairwise distances between the samples of modern genomes obtained by using the PCA analysis correlate with the pairwise geographic distances between the residential locations of the donors of genetic material. Plotting ancient genomes on PCA maps constructed in the vector space of modern genomes is a convenient tool for estimating the genetic relationship between ancient and modern humans.

In this work, we compare the results of a population ADMIXTURE analysis and PCA analysis, as well as F4-statistics values for a Bronze Age sample from the Caucasian burial ground Klady (Tsarskaya village) [19], obtained using three approaches. The following methods were used for preparing genomic libraries: 1) shotgun sequencing strategy; 2) selection of target genomic regions using the Arbor kit; 3) selection of target genomic regions using the Arbor kit with UDG pre-treatment of DNA; and 4) selection of target genomic regions using the Agilent kit with UDG pre-treatment of DNA.

## EXPERIMENTAL

**DNA isolation and genomic library preparation**

All experiments with aDNA were carried out in a clean room at the Federal Research Center "Fundamentals of Biotechnology" of the Russian Academy of Sciences (Skryabin Institute of Bioengineering). DNA isolated from an anthropological sample, namely the remains (fragments of jaws and teeth) of an adult from the Maykop culture megalithic tombs of the Bronze Age (Northwest Caucasus), was used. DNA was isolated from 100 mg of bone powder using Dabney buffer (5 M guanidine hydrochloride, 40% (v/v), isopropanol, 0.12 M sodium acetate, and 0.05% (v/v) Tween 20) and silica-coated magnetic beads [20]. The resulting DNA was used to prepare high-complexity libraries of single-stranded DNA fragments using the ACCEL-NGS 1S Plus DNA Library Kit (Swift Biosciences, USA) according to the original protocol with the following modifications: polymerase that had been developed in a way that uracil residues did not abrogate DNA synthesis (KAPA HiFi HS Uracil+RM, USA) was used at indexing PCR stages.

Three different types of DNA libraries were prepared from the same extract for next-generation sequencing. The first (I) library (KLD_SG) was obtained using shotgun sequencing of the total genome. For the second (II) library (KLD_CAP), the same preparation protocol was used, followed by enrichment of the genomic regions of interest (target enrichment). Unlike in library II, in the third library (III) (KLD_ UDG), DNA was pre-treated with a mixture of uracil-DNA glycosylase (UDG) and endonuclease VIII, which removes uracil from the DNA and converts the resulting abasic sites to single-nucleotide gaps [21]. Treatment with the mixture of UDG and endonuclease VIII successfully removes uracils in aDNA, while preserving a significant part of them at the end of fragments: the so-called "whiskers of ancientry," which are indicative of the ancient nature of DNA. Data on the fourth (IV) library type, which was synthesized from the same bone remains based on the principle of selecting target regions using the Agilent kit with pre-treatment of intact aDNA with UDG, were obtained from professor Pinhasi (University of Vienna, Austria) and designated with the index I6268 from the previously published study [22].

**Target enrichment**

The MyBaits Expert Human Affinities Prime Plus Kit [MyBaits Manual v.1.0 – Population Genomics Hybridization Capture for Target NGS, 2021. https:// arborbiosci.com/wp-content/uploads/2021/03/myBaits_Expert_HumanAffinities_v1.0_Manual.pdf] was used to capture 1.6 million SNPs from human aDNA. Reagents for enriching the selected genomic regions contained biotinylated single-strand DNA probes, which are a mixture of three probe sets: prime 1240K panel [23], Y Chr 46K (Y chromosome sites identified by the International Society of Genetic Genealogy ISOGG) and MitoTrio (probe set for three different mitochondrial genomes, including the revised Cambridge Reference Sequence (rCRS), Reconstructed Sapiens Reference Sequence (RSRS), and the Vindija Neanderthal sequence [24]). The protocol for MyBaits Expert Human Affinities Prime Plus kit includes two successive rounds of enrichment.

**Sequencing**

All three genomic libraries (both shotgun libraries and the ones enriched in the genomic regions of interest) were sequenced using the Illumina Hiseq 4000 platform (1 × 75 + 8 + 8 cycles) with single DNA reads.

**Bioinformatics analysis**

To remove contaminating DNA reads from the sequencing data, we used the BBDuk software [25] included in the BBMap package (www.sourceforge.net/ projects/bbmap/) and bacterial, fungal, plant, viral, and "others" databases (http://jgi.doe.gov/data-and-tools/bbtools/bb-tools-user-guide/). The output of the BBDuk tool was analyzed using the PALEOMIX pipeline (version 1.2.14) [26]. Sequencing adapters were trimmed using the Cutadapt v3.4 tool [27]. Sequences were aligned to the reference human genome sequence (hg19/GRCh37) using BWA (version 0.7.17) [28]. Aligned reads were filtered to provide the maximum mapping quality of 20 using the Samtools view v1.9 program [29]. Samtools v1.9 was used to index, sort, and remove duplicates (rmdup) [29].

PileupCaller (https://github.com/stschiff/sequenceTools) was used to call genotypes from the aligned reads using the "--randomHaploid" mode, which calls haploid genotypes by random selection of one high-quality base (phred base quality score ≥ 30) on the 1240K SNP panel (https://reich.hms.harvard.edu/).

Postmortem DNA damage patterns were analyzed using the MapDamage2 software [30], which offers a series of tools for imaging and modeling postmortem damage patterns observed in ancient samples. MapDamage2.0 also makes it possible to recalculate base quality scores in order to mitigate the impact of postmortem damage on further analysis.

We used the ADMIXTURE v.1.3.0 software [16] to determine the genetic clustering of a Bronze Age sample from the burial mound Klady (Caucasus) using each of the three methods of genomic library preparation, as well as other samples from the Allen Ancient DNA Resource (AADR) panel. SNPs were trimmed for sites with linkage disequilibrium using PLINK v1.9 [31]. The sliding window was 50 SNPs; the step was 5 SNPs; the r2 threshold was 0.2 (–indep-pairwise 50 5 0.2). There were 10 runs with random starting values for a number of clusters (K) in the range of 4–13; the run with the lowest cross-validation error was selected to plot the graph of population admixture.

## RESULTS AND DISCUSSION

**Characterization of genomic libraries**

The contribution of postmortem DNA modifications in the distortion of the statistical analysis results was assessed using three genomic libraries prepared from an archeological bone sample from the burial mound Klady (Tsarskaya village): (I) shotgun library KLD_SG; (II) library enriched for the target regions KLD_CAP; and (III) library enriched for the target regions and treated with the mixture of UDG and endonuclease VIII, which removes uracil residues from DNA strands and converts the resulting abasic sites to single-nucleotide gaps (KLD_UDG). Thus, we expected to find C→T substitutions in libraries I and II. These substitutions have the potential to distort the results of the genetic analysis. They are artificially deleted in library III. However, this library is expected to contain shorter fragments, which is due to UDG-mediated introduction of single-strand breaks in the original DNA.

**Table 1:** Sequencing statistics

Library	Number of input reads	Number of reads after filtration	Read average length for analysis	Number of mapped reads	After elimination of PCR duplicates	Coverage	Endogenous DNA, %	SNPs (for analysis)
KLD_SG (I)	1,473,546,011	1,469,259,287	78.02	65,025,843	46,813,163	1.17	3.18	321,229
KLD_CAP (II)	100,874,292	100,870,259	79.06	85,406,013	3,865,852	0.09	3.83	615,991
KLD_UDG (III)	58,364,547	58,329,170	63.95	52,565,836	4,392,304	0.08	7.53	690,148
I6268	*	*	*	*	1,091,304	0.81	4.02	372,480

Note. The study by Wang et al. [22] does not provide scores designated as *.

The total number of DNA reads generated for these three libraries varied from 58,364,547 to 1,473,546,011 per DNA library, while the fraction of endogenous DNA (i.e., reads mapped on human genome hg19/GRCh37) was 3.18–7.53% (Table 1).

It is important to note that evaluation of the number of SNPs suitable for analysis was conducted for the 1,2K panel used in the experiments with aDNA [32]. The number of SNPs determined for library I was almost two times lower than those in other libraries, despite the 10-fold excess of the number of reads compared to the libraries II and III.

**Fig. 1:**
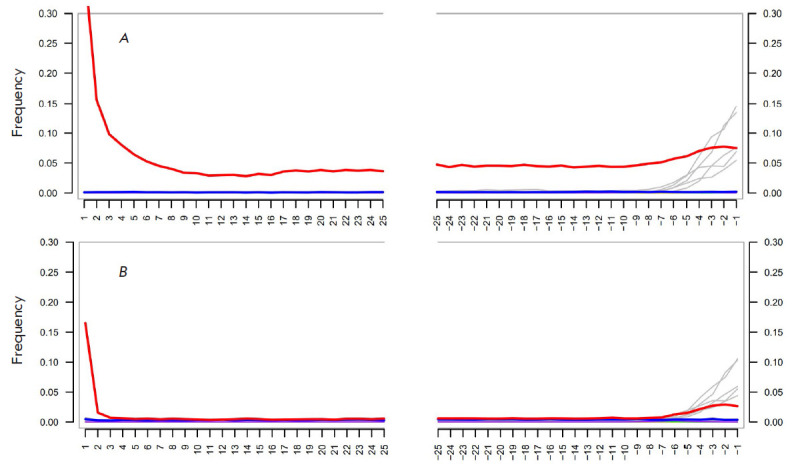
Postmortem damage patterns of DNA libraries generated using MapDamage 2.0. (*A*) – Library without UDG treatment (KLD_SG), false C→T transitions are represented by the red line (the blue line denotes complementary G→A transitions) at 5’ (positive coordinates) and 3’ ends (negative coordinates) of the last 25 nucleotides. The presence of C→T substitutions after the second reading (right half of the graph) of the complementary strand is due to the particulars of the single-strand library preparation. The drop in the deamination level for the nucleotides -5 and -1 is due to A-tailing and the experimental protocol used. (*B*) – Damage pattern in the library partially treated with UDG (KLD_UDG) and obtained from the same extract

The authenticity of the ancient DNA was determined using the MapDamage version 2.0 software, which utilizes postmortem damage patterns (Fig. 1). Taking into account that libraries treated with UDG retain a certain number of C→T substitutions at terminal 2 bp in mapped fragments, 2 bp should be deleted from both ends of the reads using trimBam from the bamUtil repository [33].

**ADMIXTURE analysis**

**Fig. 2:**
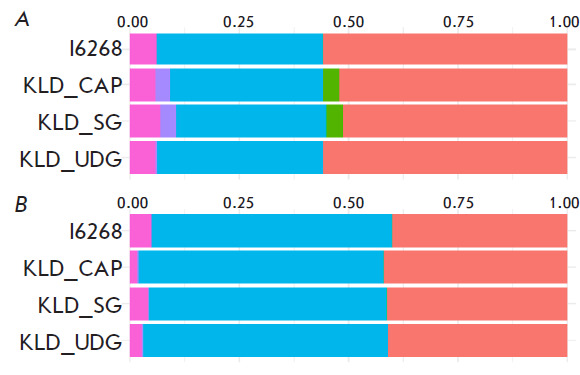
ADMIXTURE analysis (K = 7) of a genome sequenced using various methodological approaches. (*A*) – Standard admixture analysis based on David Reich’s 1240k SNP panel. Additional basic green and purple populations in the form of additional components caused by postmortem changes can be seen. (*B*) – Admixture analysis conducted for transversions only

The results of the ADMIXTURE analysis for K = 7 for three libraries prepared in this study with the previously sequenced Novosvobodnaya cultural variant sample I6268 [22], to which the Klady burials belong, demonstrated additional components in non-UDG-treated samples (samples KLD_CAP and KLD_SG; green and purple components in Fig. 2A). Based on the identity of the bone sample, the false effect of postmortem aDNA modifications on the ADMIXTURE analysis results when preparing libraries without using UDG was hypothesized.

We proposed a bioinformatics approach for reducing the negative effect of postmortem modifications. The approach is based on masking all the SNPs asso ciated with transitions (C→T and G→A). After all transitions are eliminated, ADMIXTURE analysis results for libraries not treated with UDG correlate with the analysis results for UDG-treated libraries (Fig. 2B).

**Fig. 3:**
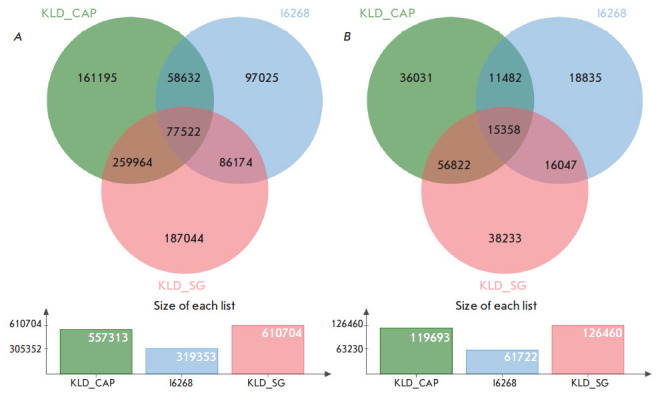
Venn diagrams for the SNPs of the three libraries (KLD_CAP, KLD_SG, and I6268). The total number of SNPs for each library is shown below the charts. (*A*) – Transversions and transitions; (*B*) – Transversions only

The fact that the proportional composition of ancestral populations changes due to the elimination of false green and purple components when analyzing transversions draws attention: the ratio of the blue component increases, while the ratios of red and pink components increase. This can be explained by the significant decrease in the number of SNPs at the input point for ADMIXTURE. Indeed, the number of transversions is approximately five times lower than the total number of SNPs. Detailed numerical parameters for all three libraries, KLD_CAP, KLD_SG, and I6268, are presented in Fig. 3.

Thus, the use of transversions in a ADMIXTURE analysis eliminates false positive signals in the form of ancestral populations that occur due to postmortal DNA modifications, but not real historical population upheavals. However, such a genetic reductionism should be used with caution, since a decrease in the total number of input data due to the exclusion of transitions can affect the reliability of the analysis results. Our empirical data show that the confidence threshold is reached when using < 30,000 SNPs.

**Fig. 4:**
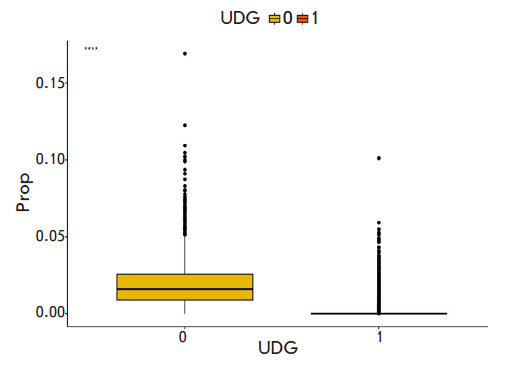
Distribution of the relative content of additional components in the ADMIXTURE analysis between samples from the Allen Ancient DNA Resource V44.3 panel treated with UDG (1) - 2,376 samples and without treatment (0) - 908 samples. Prop – the proportion of admixtures

Having analyzed all samples from the Allen Ancient DNA Resource (AADR) V44.3 panel (January 2021), we found a significant negative correlation (-0.5844) between the additional ADMIXTURE component and treatment with UDG in 3,284 European samples from the Allen Ancient DNA Resource (AADR) database (Fig. 4). It is also important to note that enrichment for target regions makes it possible to save resources during sequencing. Indeed, the generation of 58 million reads in the KLD_UDG library yields results on the structure of ancestral populations similar to those obtained using KLD_SG (~1,500 million reads).

**F4 statistics**

**Fig. 5:**
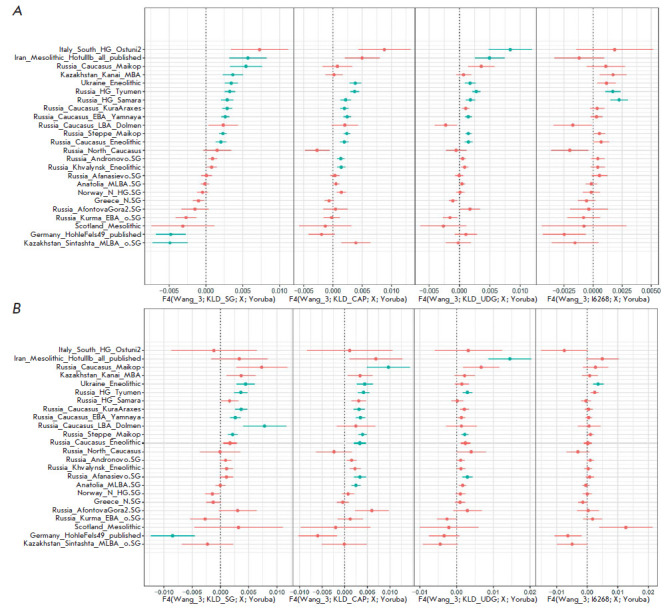
F4 statistics in the F(Wang_3, Y;X, Yoruba) configuration. The reliable metrics for |z| > 3 are colored in blue. (*A*) – F4 calculated for all SNPs from the 1240k panel. (*B*) – F4 calculated for transversions only

To study the role of sample preparation in population analysis data interpretation, we calculated the F4 statistics in the F4(Wang_3, Y;X, Yoruba) configuration. The Wang_3 population consists of three samples (I6267, I6266, and I6272) of the Novosvobodnaya cultural variant, to which sample I6268 belongs. Population X was used from a list proposed by archaeologists (provided on the left of the Y axis in Fig. 5) based on their historical hypotheses. The Yoruba population was used as the outgroup. Four SNP sets determined for KLD_ SG, KLD_CAP, KLD_UDG, and I6268 were used as Y populations. As stated above, the intuitive meaning of non-zero significant statistics will indicate which population from the X list contributes the most both to the Wang_3 population in case of positive statistics and the experimental sample Y in case of negative statistics. Interpretation of the archaeological and historical meaning of the difference between population Wang_3 and the sample used for the preparation of the four test libraries is beyond the scope of this paper. Calculations were presented only as an example to demonstrate that reliable non-zero F4 statistics can be interpreted differently depending on the method of sample preparation.

Figure 5A presents F4 statistics data for all SNPs. For KLD_SG used as Y, all populations on the right were sorted in descending order of statistical value. When using the three other libraries as Y, the sorting order changes significantly. Furthermore, reliable statistical results with the absolute z score value > 3 change: 12, 9, 8, and 2 for KLD_SG, KLD_CAP, KLD_ UDG, and I6268, respectively. Only one population from the X list, namely Russia_HG_Tyumen, is reliable for all four libraries from the Y list. However, when analyzing transversions, the sorting order for all four libraries changes compared to the original F4 sorting (Wang_3; KLD_SG; X; Yoruba). The number of reliable X populations is 7, 9, 4, and 1 for KLD_SG, KLD_CAP, KLD_UDG, and I6268, respectively. Furthermore, there is no X population that can be reliably determined using F4 statistics for all four libraries when using transversions. Figure 5B shows that even Russia_HG_Tyumen, which is the total significant population X in the analysis of all SNPs, is significant for only three libraries: KLD_SG, KLD_CAP, and KLD_UDG, but not I6268.

The conclusions of this part of the study are the following: F4 statistics is extremely sensitive to the number of input SNPs compared to ADMIXTURE, and it is important to use SNP sets obtained from uniformly prepared genomic libraries for F4 statistics. Otherwise, there is a chance of incorrect interpretation of reliable and positive absolute values of F4.

**Genetic PCA maps**

**Fig. 6:**
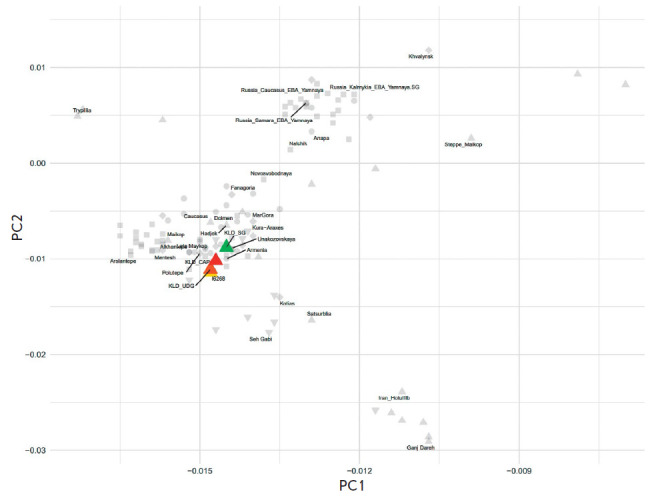
Principal component analysis (PCA). Ancient samples were projected onto the vectors of modern samples that were used only to construct PCA and are not shown in this figure

**Fig. 7:**
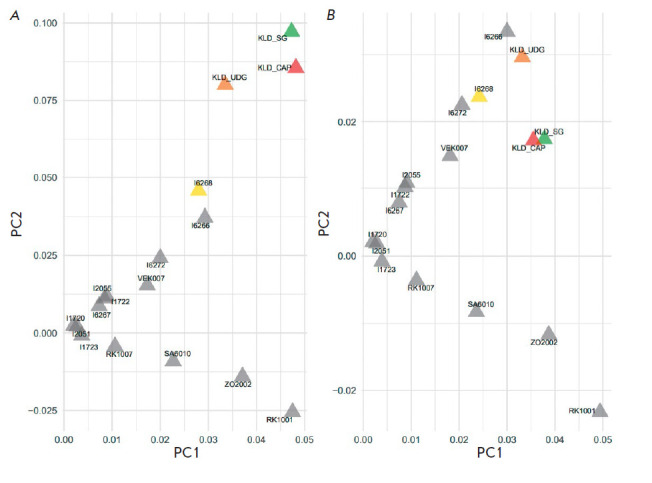
Principal component analysis (PCA) for KLD_ SG, KLD_CAP, KLD_UDG, I6268 (highlighted in color), and other Caucasian samples (shown in grey) calculated for all SNPs from the 1240k panel (*A*) and transversions only (*B*)

We also assessed the impact of sample preparation on PCA projections on the PC1–PC2 plane. A total of 253 ancient samples were used for PCA, which was first obtained for vectors of representatives of different modern Eurasian populations [34]. In order to simplify the interpretation, all ancient samples in Fig. 6 are colored in light gray, except for the four libraries studied. Samples I6268 and KLD_UDG have a minimal difference in PC1–PC2 coordinates, while KLD_SG is shifted from them in the northeast direction. To obtain a detailed idea of how the four test libraries are grouped in the analysis of both all SNPs and transver sions only, the PCA analysis was conducted using only 17 samples related historically to the Novosvobodnaya cultural variant (Fig. 7). It is important to clarify that the new PCA with 17 samples is based on the generation of new PC1–PC2 vectors which differ from those in Fig. 6. The figure shows that it is impossible to reduce even two libraries to one point on the PC1– PC2 plane when using both all SNPs (Fig. 7A) and transversions only (Fig. 7B). We see that Fst for the group KLD_SG, KLD_CAP, KLD_UDG, and I6268 is 11% when using all SNPs and 19% when using transversions only. This indicates an increase in sample concordance when using transversions, which is illustrated in a slightly tighter grouping of the four test libraries in the PCA maps.

## CONCLUSIONS

This works shows that modern statistical approaches, especially F4 statistics, are extremely sensitive to the method of sample preparation used for obtaining aDNA genomic libraries. We consider the selection of target regions with pre-treatment of aDNA with UDG as the optimal approach to generating genomic libraries. Even with this approach, the use of enrichment kits from different manufacturers can generate false positive results in the statistical analysis. In order to minimize the effect of the methodological approaches, we recommend increasing the expeditionary samples of bone remains of representatives of the same culture/population and, if possible, consolidating sample preparation in one study.

## Abbreviations

UDG: uracil-DNA glycosylase
aDNA: ancient DNA
SNP: single nucleotide polymorphism
PCA: principal component analysis
